# On *F*-Algebras *M* ^*p*^  (1 < *p* < ∞) of Holomorphic Functions

**DOI:** 10.1155/2014/901726

**Published:** 2014-01-28

**Authors:** Romeo Meštrović

**Affiliations:** Maritime Faculty, University of Montenegro, Dobrota 36, 85330 Kotor, Montenegro

## Abstract

We consider the classes *M* ^*p*^ (1 < *p* < ∞) of holomorphic functions on the open unit disk **𝔻** in the complex plane. These classes are in fact generalizations of the class *M* introduced by Kim (1986). The space *M* ^*p*^ equipped with the topology given by the metric **ρ**_*p*_ defined by **ρ**_*p*_(*f*, *g*) = ||*f* − *g*||_*p*_ = (*∫*_0_^2**π**^log^*p*^(1 + *M*(*f* − *g*)(**θ**))(*d*θ**/2**π**))^1/*p*^, with *f*, *g∈M* ^*p*^ and *Mf*(**θ**) = sup_0*⩽*r<1_ *⁡*|*f*(*re* ^*i*θ**^)|, becomes an *F*-space. By a result of Stoll (1977), the Privalov space *N* ^*p*^ (1 < *p* < ∞) with the topology given by the Stoll metric *d*_*p*_ is an *F*-algebra. By using these two facts, we prove that the spaces *M* ^*p*^ and *N* ^*p*^ coincide and have the same topological structure. Consequently, we describe a general form of continuous linear functionals on *M* ^*p*^ (with respect to the metric **ρ**_*p*_). Furthermore, we give a characterization of bounded subsets of the spaces *M* ^*p*^. Moreover, we give the examples of bounded subsets of *M* ^*p*^ that are not relatively compact.

## 1. Introduction and Preliminaries

Let *𝔻* denote the open unit disk in the complex plane and let *𝕋* denote the boundary of *𝔻*. Let *L* ^*q*^(*𝕋*)  (0 < *q* ≤ *∞*) be the familiar Lebesgue spaces on the unit circle *𝕋*.

Following Kim ([1, 2]), the class  *M* consists of all holomorphic functions *f* on *𝔻* for which

(1) $$\begin{array}{c} \int_{0}^{2\pi }\log^{+}Mf\left(\theta \right)\frac{d\theta }{2\pi }<\infty , \end{array}$$

where log⁡^+^⁡|*a*| = max⁡⁡{log⁡⁡*a*, 0} and

(2) $$\begin{array}{c} Mf\left(\theta \right)=\underset{0⩽r<1}{\sup}\left|f\left(re^{i\theta }\right)\right| \end{array}$$

is the maximal radial function of *f*. The Privalov class  *N* ^*p*^  (1 < *p* < *∞*) consists of all holomorphic functions *f* on *𝔻* for which

(3) $$\begin{array}{c} \underset{0<r<1}{\sup}\int_{0}^{2\pi }{\left(\log^{+}\left|f\left(re^{i\theta }\right)\right|\right)}^{p}\frac{d\theta }{2\pi }<+\infty . \end{array}$$

These classes were firstly considered by Privalov in [3, page 93], where *N* ^*p*^ is denoted as *A*_*q*_.

Notice that for *p* = 1, the condition (3) defines the Nevanlinna class  *N* of holomorphic functions in *𝔻*. Recall that the Smirnov class  *N* ^+^ is the set of all functions *f* holomorphic on *𝔻* such that

(4) $$\begin{array}{c} \underset{r\to 1}{\lim}\int_{0}^{2\pi }\log^{+}\left|f\left(re^{i\theta }\right)\right|\frac{d\theta }{2\pi }=\int_{0}^{2\pi }\log^{+}\left|f^{*}\left(e^{i\theta }\right)\right|\frac{d\theta }{2\pi }<+\infty , \end{array}$$

where f* is the boundary function of *f* on *𝕋*; that is,

(5) $$\begin{array}{c} f^{*}\left(e^{i\theta }\right)=\underset{r\to 1-}{\lim}f\left(re^{i\theta }\right) \end{array}$$

is the radial limit of *f* which exists for almost every *e* ^*iθ*^. We denote by *H* ^*q*^  (0 < *q* ≤ *∞*) the classical Hardy space on *𝔻*. It is known (see [4, 5]) that

(6) $$\begin{array}{c} N^{r}\subset N^{p}\;\left(r>p\right),\;\;\underset{q>0}{⋃}H^{q}\subset \underset{p>1}{⋂}N^{p},\\ \underset{p>1}{⋃}N^{p}\subset M\subset N^{+}\subset N, \end{array}$$

where the above containment relations are proper.

The study of the spaces *N* ^*p*^  (1 < *p* < *∞*) was continued in 1977 by Stoll [6] (with the notation (log⁡^+^ *H*)^*α*^ in [6]). Further, the topological and functional properties of these spaces were studied in [4, 5, 7–14]; typically, the notation varied and these spaces are called the Privalov spaces in [12–15].

It is well known [16, page 26] that a function *f* ∈ *N* ^+^ if and only if *f* = *IF*, where *I* is an inner function on *𝔻* and *F* is an outer function given by

(7) $$\begin{array}{c} F\left(z\right)=\exp\left(\int_{0}^{2\pi }\frac{e^{it}+z}{e^{it}-z}\log\left|F^{*}\left(e^{it}\right)\right|\frac{dt}{2\pi }\right), \end{array}$$

where log⁡⁡|F*| ∈ *L* ^1^(*𝕋*).

Privalov [3, page 98] showed that *f* ∈ *N* ^*p*^ if and only if *f* = *IF*, where *I* is an inner function on *𝔻* and *F* is an outer function as given above with log⁡^+^ | f* | ∈*L* ^*p*^(*𝕋*).

Stoll [6, Theorem 4.2] showed that the space *N* ^*p*^ (with the notation (log⁡^+^ *H*)^*α*^ in [6]) with the topology given by the metric *d*_*p*_ defined by

(8) dp(f,g)=(∫02π(log⁡⁡(1+|f∗(eiθ)−g∗(eiθ)|))pdθ2π)1/p,f,g∈Np

becomes an *F*-algebra. Recall that the function *d*_1_ = *d* defined on the Smirnov class *N* ^+^ by (8) with *p* = 1 induces the metric topology on *N* ^+^. Yanagihara [17] showed that, under this topology, *N* ^+^ is an *F*-space.

Furthermore, in connection with the spaces *N* ^*p*^  (1 < *p* < *∞*), Stoll [6] (also see [7] and [12, Section 3]) also studied the spaces *F* ^*q*^  (0 < *q* < *∞*) (with the notation *F*_1/*q*_ in [6]), consisting of those functions *f* holomorphic on *𝔻* for which

(9) $$\begin{array}{c} \underset{r\to 1}{\lim}{\left(1-r\right)}^{1/q}\log^{+}M_{\infty }\left(r,f\right)=0, \end{array}$$

where

(10) $$\begin{array}{c} M_{\infty }\left(r,f\right)=\underset{\left|z\right|\leq r}{\max}\left|f\left(z\right)\right|. \end{array}$$

Stoll [6, Theorem 3.2] proved that the space *F* ^*q*^ with the topology given by the family of seminorms {||·||_*q*,*c*_}_*c*>0_ defined for *f* ∈ *F* ^*q*^ as

(11) $$\begin{array}{c} {\left|||f||\right|}_{q,c}=\sum_{n=0}^{\infty }\left|\hat{f}\left(n\right)\right|e^{-cn^{1/(q+1)}}<\infty , \end{array}$$

for each *c* > 0, where $\hat{f}(n)$ is the *n*th Taylor coefficient of *f*, becomes a countably normed Fréchet algebra. By a result of Eoff [7, Theorem 4.2], *F* ^*p*^ is the Fréchet envelope  of  *N* ^*p*^, and hence *F* ^*p*^ and *N* ^*p*^ have the same topological duals.

Here, as always in the sequel, we will need some of Stoll's results concerning the spaces *F* ^*q*^ only with 1 < *q* < *∞*, and hence we will assume that *q* = *p* > 1 is any fixed number.

The study of the class *M* has been extensively investigated by Kim in [1, 2], Gavrilov and Zaharyan [18], and Nawrocky [19]. Kim [2, Theorems 3.1 and 6.1] showed that the space *M* with the topology given by the metric *ρ* defined by

(12) $$\begin{array}{c} \rho \left(f,g\right)=\int_{0}^{2\pi }\log\left(1+M\left(f-g\right)\left(\theta \right)\right)\frac{d\theta }{2\pi },\;f,g\in M \end{array}$$

becomes an *F*-algebra. Furthermore, Kim [2, Theorems 5.2 and 5.3] gave an incomplete characterization of multipliers of *M* into *H* ^*∞*^. Consequently, the topological dual of *M* is not exactly determined in [2], but, as an application, it was proved in [2, Theorem 5.4] (also cf. [19, Corollary 4]) that *M* is not locally convex space. Furthermore, the space *M* is not locally bounded ([2, Theorem 4.5] and [19, Corollary 5]).

Although the class *M* is essentially smaller than the class *N* ^+^, Nawrocky [19] showed that the class *M* and the Smirnov class *N* ^+^ have the same corresponding locally convex structure which was already established by Yanagihara for the Smirnov class in [17, 20]. More precisely, it was proved in [19, Theorem 1] that the Fréchet envelope of the class *M* can be identified with the space *F* ^+^ of holomorphic functions on the open unit disk *𝔻* such that

(13) $$\begin{array}{c} {\left|||f||\right|}_{c}:=\sum_{n=0}^{\infty }\left|\hat{f}\left(n\right)\right|e^{-c\sqrt{n}}<\infty , \end{array}$$

for each *c* > 0, where $\hat{f}(n)$ is the *n*th Taylor coefficient of *f*. Notice that *F* ^+^ coincides with the space *F* ^1^ defined above. It was shown in [17, 21] that *F* ^+^ is actually the containing Fréchet space for *N* ^+^. Moreover, Nawrocky [19, Theorem 1] characterized the set of all continuous linear functionals on *M* which by a result of Yanagihara [17] coincides with those on the Smirnov class *N* ^+^.

Motivated by the mentioned investigations of the classes *M* and *N* ^+^, and the fact that the classes *N* ^*p*^  (1 < *p* < *∞*) are generalizations of the Smirnov class *N* ^+^, in Section 2, we consider the classes *M* ^*p*^  (1 < *p* < *∞*) as generalizations of the class *M*. Accordingly, the * class* *M* ^*p*^  (1 < *p* < *∞*) consists of all holomorphic functions *f* on *𝔻* for which

(14) $$\begin{array}{c} \int_{0}^{2\pi }{\left(\log^{+}Mf\left(\theta \right)\right)}^{p}\frac{d\theta }{2\pi }<\infty . \end{array}$$

Obviously,

(15) $$\begin{array}{c} \overset{}{\underset{p>1}{⋃}}M^{p}\subset M. \end{array}$$

Following [2], by analogy with the space *M*, the space *M* ^*p*^ is equipped with the topology induced by the metric *ρ*_*p*_ defined as

(16) ρp(f,g)=||f−g||p=(∫02πlog⁡p⁡(1+M(f−g)(θ))dθ2π)1/p,

with *f*, *g* ∈ *M* ^*p*^.

In Section 2, we give the integral limit criterion for a function *f* holomorphic on the disk *𝔻* to belong to the class *M* ^*p*^ (Lemma 3). Furthermore, we prove that the space *M* ^*p*^ is closed under integration (Theorem 4).

In Section 3 we study and compare the uniform convergence on compact subsets of *𝔻* and the convergences induced by the metrics *ρ*_*p*_ and *d*_*p*_ in the space *M* ^*p*^, respectively. It is proved (Theorem 11) that *M* ^*p*^ = *N* ^*p*^ for each *p* > 1.

It is proved in Section 4 that the space of all polynomials on *ℂ* is a dense subset of *M* ^*p*^ (Theorem 13). Hence, *M* ^*p*^ is a separable metric space. We show that the space *M* ^*p*^ with the topology given by the metric *ρ*_*p*_ becomes an *F*-space (Theorem 15). As an application, we prove that the metric spaces (*M* ^*p*^, *ρ*_*p*_) and (*N* ^*p*^, *d*_*p*_) have the same topological structure (Theorem 16). Consequently, we obtain a characterization of continuous linear functionals on *M* ^*p*^ (Theorem 17). Notice that Theorem 17 with *p* = 1 characterizes the set of all continuous linear functionals on the space *M*, which is in fact the Nawrocky result [19, Theorem 1] mentioned above.

In Section 5 we obtain a characterization of bounded subsets of the spaces *M* ^*p*^( = *N* ^*p*^) (Theorem 19). It is also given another necessary condition for a subset of *M* ^*p*^ (*N* ^*p*^) to be bounded (Theorem 22). Finally, we give the examples of bounded subsets of *M* ^*p*^ that are not relatively compact (Theorem 24).

## 2. The Classes *M* ^*p*^  (1 < *p* < *∞*)

Recall that, for a fixed 1 < *p* < *∞*, the class *M* ^*p*^ consists of all holomorphic functions *f* on *𝔻* for which

(17) $$\begin{array}{c} \int_{0}^{2\pi }{\left(\log^{+}Mf\left(\theta \right)\right)}^{p}\frac{d\theta }{2\pi }<\infty . \end{array}$$

Combining the inequalities log⁡(|*a* | +1) ≤ log⁡^+^ | *a* | +log⁡2 and (|*b* | +|*c* | )^*p*^ ≤ 2^*p*−1^(|*b*|^*p*^ + |*c*|^*p*^), we obtain log⁡^*p*^(|*a* | +1) ≤ 2^*p*−1^((log⁡^+^ | *a* | )^*p*^ + (log⁡2)^*p*^)  (*a*, *b*, *c* ∈ *ℂ*). The last inequality implies the fact that the condition (17) is equivalent to

(18) $$\begin{array}{c} {||f||}_{p}:={\left(\int_{0}^{2\pi }{\left(\log\left(1+Mf\left(\theta \right)\right)\right)}^{p}\frac{d\theta }{2\pi }\right)}^{1/p}<\infty . \end{array}$$

**Lemma 1**

The function ||·||_*p*_ defined on *M* ^*p*^ by (18) satisfies the following conditions:

(19) $$\begin{array}{c} {||f+g||}_{p}\leq {||f||}_{p}+{||g||}_{p}\;\forall f,g\in M^{p}, \end{array}$$

(20) $$\begin{array}{c} {||fg||}_{p}\leq {||f||}_{p}+{||g||}_{p}\;\forall f,g\in M^{p}. \end{array}$$

Hence, *M* ^*p*^ is an algebra with respect to the pointwise addition and multiplication of functions.

**Proof**

Combining the inequality

(21) log⁡⁡(1+M(f+g)(θ)) ≤log⁡⁡(1+Mf(θ))+log⁡⁡(1+Mg(θ)),                f,g∈Mp

with Minkowski's integral inequality (with the power *p*), we immediately obtain (19). Similarly, combining the inequality

(22) log⁡⁡(1+M(fg)(θ)) ≤log⁡⁡(1+Mf(θ))+log⁡⁡(1+Mg(θ)),                f,g∈Mp

with Minkowski's integral inequality (with the exponent *p*), we obtain (20).

**Theorem 2**

The function *ρ*_*p*_ defined on *M* ^*p*^ as

(23) ρp(f,g)=||f−g||p=(∫02πlog⁡p⁡(1+M(f−g)(θ))dθ2π  )1/p,                f,g∈Mp

is a translation invariant metric on *M* ^*p*^. Further, the space *M* ^*p*^ is a complete metric space with respect to the metric *ρ*_*p*_.

**Proof**

If we suppose that *ρ*_*p*_(*f*, *g*) = 0, for some *f*, *g* ∈ *M* ^*p*^, then by (23) it follows that *M*(*f* − *g*)(*θ*) = 0 for almost every *θ* ∈ [0,2*π*]. Hence, f*(*e* ^*iθ*^) = g*(*e* ^*iθ*^) for almost every *e* ^*iθ*^ ∈ *𝕋*, and, by Riesz uniqueness theorem, we infer that *f*(*z*) = *g*(*z*) for all *z* ∈ *𝔻*. As, by (19), the triangle inequality is satisfied, it follows that *ρ*_*p*_ is a metric on *M* ^*p*^. Finally, by the obvious inequality

(24) $$\begin{array}{c} \rho_{p}\left(f+h,g+h\right)=\rho_{p}\left(f,g\right),\;f,g,h\in M^{p}, \end{array}$$

we see that *ρ*_*p*_ is a translation invariant metric. This concludes the proof.

For simplicity, here as always in the sequel, we shall write *M* ^*p*^ instead of the metric space (*M* ^*p*^, *ρ*_*p*_). For a function *f* holomorphic in *𝔻* and for any fixed 0 ≤ *ρ* < 1, denote by *f*_*ρ*_ the function defined on *𝔻* as *f*_*ρ*_(*z*) = *f*(*ρz*), *z* ∈ *𝔻*. Furthermore, for a given holomorphic function *f* on *𝔻*, let

(25) $$\begin{array}{c} {Mf}_{\rho }\left(\theta \right)=\underset{0\leq r\leq \rho }{\sup}\left|f_{r}\left(\theta \right)\right|=\underset{0\leq r\leq \rho }{\sup}\left|f\left(re^{i\theta }\right)\right|,\;0\leq \rho <1. \end{array}$$

**Lemma 3**

A function *f* holomorphic on the unit disk *𝔻* belongs to the class *M* ^*p*^ if and only if it satisfies

(26) lim⁡ρ→1⁡∫02π(log⁡+Mfρ(θ))pdθ2π =∫02π(log⁡+Mf(θ))pdθ2π<∞.

**Proof**

The condition (26) implies that *f* ∈ *M* ^*p*^. Conversely, assume that *f* ∈ *M* ^*p*^. Then

(27) Mfρ(θ)⟶Mf(θ)  as  ρ⟶1  for almost every  θ∈[0,2π].

Since, by the assumption, *f* ∈ *M* ^*p*^; that is, ∫_0_^2*π*^(log⁡^+^ *Mf*(*θ*))^*p*^(*dθ*/2*π*) < *∞*, using (27) and applying the Lebesgue dominated convergence theorem, we obtain

(28) $$\begin{array}{c} \underset{\rho \to 1}{\lim}\int_{0}^{2\pi }{\left(\log^{+}Mf_{\rho }\left(\theta \right)\right)}^{p}\frac{d\theta }{2\pi }=\int_{0}^{2\pi }{\left(\log^{+}Mf\left(\theta \right)\right)}^{p}\frac{d\theta }{2\pi }\;, \end{array}$$

which completes the proof.

**Theorem 4**

The space *M* ^*p*^ is closed under integration.

**Proof**

For a given function *f* ∈ *M* ^*p*^, define

(29) $$\begin{array}{c} F\left(z\right)=\int_{0}^{z}f\left(z\right)dz=\int_{0}^{r}f\left({te}^{i\theta }\right)e^{i\theta }dt. \end{array}$$

It follows that |*F*(*re* ^*iθ*^)|≤*Mf*(*θ*), and thus *MF*(*θ*) ≤ *Mf*(*θ*) for almost every *θ* ∈ [0,2*π*]. Therefore *F* ∈ *M* ^*p*^, as desired.

## 3. Convergences in the Space *M* ^*p*^

**Theorem 5**

For each function *f* ∈ *M* ^*p*^, *f*_*ρ*_ → *f* in *M* ^*p*^ as *ρ* → 1−.

**Proof**

Assume that *f* ∈ *M* ^*p*^. Since *f* ∈ *N* ^+^, by Fatou's theorem, the radial limit f*(*e* ^*iθ*^) = lim⁡_*r*→1−_ *f*(*re* ^*iθ*^) exists for almost every *θ* ∈ [0,2*π*]. Hence, for such a fixed *θ*, the function *t* ↦ *f*(*te* ^*iθ*^) is a continuous on [0,1], and thus it is uniformly continuous on [0,1]. Therefore, for such a *θ*, we have

(30) $$\begin{array}{c} M\left(f-f_{\rho }\right)\left(\theta \right)⟶0\;\text{as}\;\;\rho ⟶1-. \end{array}$$

By the inequality

(31) log⁡⁡(1+M(f−fρ)(θ)) ≤log⁡⁡(1+Mf(θ))+log⁡⁡(1+Mfρ(θ)) ≤2log⁡⁡(1+Mf(θ)),

in view of the fact that (18) is satisfied for *f* ∈ *M* ^*p*^, we obtain

(32) $$\begin{array}{c} \log^{p}\left(1+M\left(f-f_{\rho }\right)\left(\theta \right)\right)\leq 2^{p}\log^{p}\left(1+Mf\left(\theta \right)\right)\in L^{1}\left(𝕋\right). \end{array}$$

From this and (30), by the Lebesgue dominated convergence theorem, we obtain

(33) ∫02π(log⁡⁡(1+M(f−fρ)(θ)))pdθ2π⟶0,as  ρ⟶1−.

That is, *f*_*ρ*_ → *f* in *M* ^*p*^ as *ρ* → 1−.

For the proof of completeness of the metric space (*M* ^*p*^, *ρ*_*p*_) we will need the following lemmas.

**Lemma 6**

If {*f*_*n*_} is a Cauchy sequence in *M* ^*p*^, then (*f*_*n*_)_*ρ*_ → *f*_*n*_ in *M* ^*p*^ as *ρ* → 1−, where this convergence is uniform with respect to *n* ∈ *ℕ*.

**Proof**

Suppose that {*f*_*n*_} is an arbitrary Cauchy sequence in *M* ^*p*^. Then for a given *ε* > 0 there is a *k* ∈ *ℕ* such that

(34) $$\begin{array}{c} \rho_{p}\left(f_{n},f_{m}\right)<\frac{\varepsilon }{3}\;\forall n,m\geq k. \end{array}$$

So by the triangle inequality, for each *n* ≥ *k*, we have

(35) ρp(fn,(fn)ρ)≤ρp(fn,fk)+ρp(fk,(fk)ρ) +ρp((fk)ρ,(fn)ρ)≤2ρp(fn,fk)+ρp(fk,(fk)ρ)<2ε3+ρp(fk,(fk)ρ).

By Theorem 5, there exists 0 < *ρ*_0_ < 1  sufficiently near to 1, for which

(36) ρp(fl,(fl)ρ)<ε3 for each  ρ0<ρ<1,for each  l=1,…,k.

Hence, by (35), we immediately obtain

(37) ρp(fn,(fn)ρ)<ε for each   ρ0<ρ<1,  for each  n∈ℕ.

This completes proof of Lemma 6.

**Lemma 7**

For any *p* > 1, *M* ^*p*^⊆*N* ^*p*^ and

(38) $$\begin{array}{c} d_{p}\left(f,g\right)\leq \rho_{p}\left(f,g\right)\;for\;\;each\;\;f,g\in M^{p}, \end{array}$$

where *d*_*p*_ is the metric of *N* ^*p*^ defined by (8).

**Proof**

The inclusion *M* ^*p*^⊆*N* ^*p*^ is obvious, and (38) follows by the definition of the metrics *d*_*p*_ and *ρ*_*p*_.

**Lemma 8**

The convergence with respect to the metric *d*_*p*_ of the space *N* ^*p*^ is stronger than the metric of uniform convergence on compact subsets of the disk *𝔻*.

**Proof**

The assertion immediately follows from the inequality on [5, page 898], which implies that, for any function *f* ∈ *N* ^*p*^ and 0 ≤ *r* < 1, we have

(39) $$\begin{array}{c} \underset{\left|z\right|=r}{\max}\left|f\left(z\right)\right|\leq \exp\left({\left(\frac{1+r}{1-r}\right)}^{1/p}d_{p}\left(f,0\right)\right). \end{array}$$

**Lemma 9**

If {*f*_*n*_} is a Cauchy sequence in the space *M* ^*p*^, then {*f*_*n*_} converges uniformly on compact subsets of *𝔻* to some holomorphic function *f* on *𝔻*.

**Proof**

From the inequality (38) of Lemma 7, it follows that {*f*_*n*_} is a Cauchy sequence in *N* ^*p*^. Therefore, there exists *f* ∈ *N* ^*p*^ such that *f*_*n*_ → *f* in *N* ^*p*^, and so, by Lemma 8, *f*_*n*_ → *f* uniformly on compact subsets of *𝔻*.

The following result is a maximal theorem of Hardy and Littlewood.

**Lemma 10 (see [16, page 11])**

Let 1 < *p* ≤ +*∞* and let *φ* be a function in the Lebesgue space *L* ^*p*^(*𝕋*). Let

(40) $$\begin{array}{c} u\left(r,\theta \right)=\frac{1}{2\pi }\int_{0}^{2\pi }\frac{1-r^{2}}{1-2r\cos\left(\theta -t\right)+r^{2}}\varphi \left(t\right)dt,\;0\leq r<1 \end{array}$$

be the Poisson integral of the function *φ*. Define

(41) $$\begin{array}{c} U\left(\theta \right)=\underset{0\leq r<1}{\sup}\left|u\left(r,\theta \right)\right|,\;\theta \in \left[0,2\pi \right]. \end{array}$$

Then *U* ∈ *L* ^*p*^(*𝕋*) and there is a constant *A*_*p*_ depending only on *p* such that

(42) $$\begin{array}{c} {||U||}_{L^{p}}\leq A_{p}{||\varphi ||}_{L^{p}}, \end{array}$$

where ||·||_*L*^*p*^_ is the usual norm of the space *L* ^*p*^(*𝕋*).

We are now ready to state the following result.

**Theorem 11**

*M* ^*p*^ = *N* ^*p*^ for each *p* > 1; that is, the spaces *M* ^*p*^ and *N* ^*p*^ coincide.

**Proof**

By Lemma 7, *M* ^*p*^⊆*N* ^*p*^ for each *p* > 1. For the proof of the converse of this inclusion, assume that *f* ∈ *N* ^*p*^. We will show that *f* ∈ *M* ^*p*^. As noticed in Section 1, *f* can be factorized as

(43) $$\begin{array}{c} f\left(z\right)=I\left(z\right)F\left(z\right),\;z\in 𝔻, \end{array}$$

where *I*(*z*) is the inner function and *F*(*z*) is an outer function for the class *N* ^*p*^; that is,

(44) $$\begin{array}{c} F\left(z\right)=\omega \exp\left(\frac{1}{2\pi }\int_{0}^{2\pi }\frac{e^{it}+z}{e^{it}-z}\log\left|f^{*}\left(e^{it}\right)\right|dt\right), \end{array}$$

where *ω* is a constant of unit modulus. Furthermore, log⁡^+^ | f* | ∈*L* ^*p*^(*𝕋*). As |*I*(*z*)|≤1, for each *z* ∈ *𝔻*, the previous factorization and the fact that *F* ∈ *M* ^*p*^ immediately imply that *f* ∈ *M* ^*p*^. Since

(45) $$\begin{array}{c} ℜ\frac{e^{it}+z}{e^{it}-z}=\frac{1-r^{2}}{1-2r\cos\left(\theta -t\right)+r^{2}},\;z=re^{i\theta }, \end{array}$$

from (44), we immediately obtain

(46) log⁡⁡|F(reiθ)| =12π∫02π1−r21−2rcos⁡⁡(θ−t)+r2log⁡⁡|f∗(eit)|dt,                    0≤r<1,

whence it follows that, for 0 ≤ *r* < 1,

(47) log⁡+⁡|F(reiθ)| =(12π∫02π1−r21−2rcos⁡(θ−t)+r2log⁡⁡|f∗(eit)|dt)+ ≤12π∫02π1−r21−2rcos⁡⁡(θ−t)+r2log⁡+⁡|f∗(eit)|dt.

The above inequality yields

(48) log⁡+⁡MF(θ)  ≤sup⁡0≤r<1⁡(log⁡+⁡|F(reiθ)|)≤sup⁡0≤r<1(12π∫02π1−r21−2rcos⁡⁡(θ−t)+r2    ×log⁡+⁡|f∗(eit)|dt).

From the above inequality and the fact that log⁡^+^ | f* | ∈*L* ^*p*^(*𝕋*), we conclude by Lemma 10 that log⁡^+^ *MF*(*θ*) ∈ *L* ^*p*^(*𝕋*). This means that *F* ∈ *M* ^*p*^ and therefore *f* ∈ *M* ^*p*^. Thus *N* ^*p*^⊆*M* ^*p*^, and therefore *M* ^*p*^ = *N* ^*p*^. This completes the proof.

**Corollary 12**

Let *f* ∈ *M* ^*p*^. Then

(49) ∫02π(log⁡+Mf(θ))p  dθ ≤Cp∫02π(log⁡+⁡|f∗(eiθ)|)pdθ,

where *C*_*p*_ is a nonnegative constant depending only on *p*.

**Proof**

Let *F* be the outer factor in the canonical factorization of *f* ∈ *M* ^*p*^. From the proof of Theorem 11, we see that for the functions *U*(*θ*) = log⁡^+^ *MF*(*θ*) and *φ*(*θ*) = log⁡^+^ | f*(*e* ^*iθ*^)| the inequality (42) can be applied from Lemma 10. The obtained inequality is in fact (49) with *F* instead of *f*. Since |*f*(*z*)|≤|*F*(*z*)|, for each *z* ∈ *𝔻*, it follows that *Mf*(*θ*) ≤ *MF*(*θ*) at almost every *θ* ∈ [0,2*π*]; thus (49) is obviously satisfied.

## 4. *M* ^*p*^ as an *F*-Algebra

**Theorem 13**

The space of all polynomials over *ℂ* is a dense subset of *M* ^*p*^. Hence, *M* ^*p*^ is a separable metric space.

**Proof**

Suppose that *f* ∈ *M* ^*p*^. Since, for a fixed 0 ≤ *ρ* < 1, *f*_*ρ*_ is a holomorphic function on the closed unit disk $\bar{𝔻}:|z|\leq 1$, by Runge's theorem, *f*_*ρ*_ can be uniformly approximated by polynomials on $\bar{𝔻}$. This together with the fact that, by Theorem 5, *f*_*ρ*_ → *f* in *M* ^*p*^ as *ρ* → 1− yields that the space of all polynomials over *ℂ* is a dense subset of *M* ^*p*^. Therefore, the set of all polynomials whose coefficients have rational real parts and rational imaginary parts becomes a countable dense subset of *M* ^*p*^. This concludes the proof.

**Theorem 14**

*M* ^*p*^ is a complete metric space.

**Proof**

Let {*f*_*n*_} be a Cauchy sequence in *M* ^*p*^. Then since *N* ^*p*^ is complete, there is a *f* ∈ *N* ^*p*^ such that *f*_*n*_ → *f* in *N* ^*p*^. Since, by Theorem 11, *M* ^*p*^ = *N* ^*p*^, it follows that *f* ∈ *M* ^*p*^, and thus it remains to show that *f*_*n*_ → *f* in *M* ^*p*^. By Theorem 5 and Lemma 6, there exist 0 < *r* < 1 and *n*_1_ ∈ *ℕ* such that

(50) $$\begin{array}{c} \rho_{p}\left(f_{r},f\right)<\frac{\varepsilon }{3},\\ \rho_{p}\left(f_{n},{\left(f_{n}\right)}_{r}\right)<\frac{\varepsilon }{3}\;\text{for each}\;\;n\geq n_{1}. \end{array}$$

Since, by Lemma 9, a sequence {*f*_*n*_} converges uniformly on each closed disk |*z* | ≤*ρ* < 1 to some function *f*, it follows that there exists *n*_2_ ∈ *ℕ* such that

(51) $$\begin{array}{c} \rho_{p}\left({\left(f_{n}\right)}_{r},f_{r}\right)<\frac{\varepsilon }{3}\;\text{for each}\;\;n\geq n_{2}. \end{array}$$

Taking *n*_0_ = max⁡{*n*_1_, *n*_2_}, by (50) and (51), the triangle inequality implies that

(52) $$\begin{array}{c} \rho_{p}\left(f_{n},f\right)<\varepsilon \;\forall n\geq n_{0}. \end{array}$$

This shows that *f*_*n*_ → *f* in *M* ^*p*^, which completes the proof.

**Theorem 15**

*M* ^*p*^ with the topology given by the metric *ρ*_*p*_ defined by (23) becomes an *F*-space.

**Proof**

By [22, page 51], it suffices to show the following properties:

- *ρ*_*p*_ is an additive-invariant metric,
- for any fixed *f* ∈ *M* ^*p*^, *c* ↦ *cf* is a continuous map from *ℂ* into *M* ^*p*^,
- for any fixed *c* ∈ *ℂ*, *f* ↦ *cf* is a continuous map from *M* ^*p*^ into *M* ^*p*^, and
- *M* ^*p*^ is a complete metric space.

The assertion (i) follows from Theorem 2.

By the Lebesgue dominated convergence theorem, we have

(53) ρp(cf,0)=(∫02πlog⁡p⁡(1+|c|Mf(θ))dθ2π)1/p⟶0as  c⟶0.

Let *k* ∈ *ℕ* such that |*c*| ≤ *k*. Then the triangle inequality yields

(54) $$\begin{array}{c} \rho_{p}\left(cf,0\right)\leq \rho_{p}\left(kf,0\right)\leq k\rho_{p}\left(f,0\right), \end{array}$$

whence we see that *f* ↦ *cf* is a continuous map from *M* ^*p*^ into *M* ^*p*^.

The assertion (iv) is in fact the assertion of Theorem 14.

This concludes the proof.

We are now ready to prove that the (metric) spaces (*M* ^*p*^, *ρ*_*p*_) and (*N* ^*p*^, *d*_*p*_) have the same topological structure.

**Theorem 16**

For each *p* > 1, the classes *M* ^*p*^ and *N* ^*p*^ coincide, and the metric spaces (*M* ^*p*^, *ρ*_*p*_) and (*N* ^*p*^, *d*_*p*_) have the same topological structure.

**Proof**

Consider the identity map *j* : *M* ^*p*^ → *N* ^*p*^. Then, by the inequality (38) of Lemma 7, *j* is continuous. Since, by Theorem 11, *M* ^*p*^ = *N* ^*p*^, *j* maps *M* ^*p*^ onto *N* ^*p*^. Since *M* ^*p*^ and *N* ^*p*^ are both *F*-spaces, it follows, by the open mapping theorem [23, Corollary 2.12 (b)], that the inverse map *j* ^−1^ of *j* is continuous. Hence, *j* is a homeomorphism, and so the metrics *d*_*p*_ and *ρ*_*p*_ induce the same topology on *N* ^*p*^ and *M* ^*p*^, respectively.

As an application of Theorem 16 and using the characterization of topological dual of the space *F* ^*p*^ (which is by [7, Theorem 4.2] the Fréchet envelope of *N* ^*p*^) given by Stoll [6, Theorem 3.3] (cf. also [12, Theorem 3.5] and [13, Theorem 2]), we immediately get the following result.

**Theorem 17**

If *γ* is a continuous linear functional on *M* ^*p*^, then there exists a sequence {*γ*_*n*_}_*n*_ of complex numbers with *γ*_*n*_ = *O*(exp⁡(−*cn* ^1/(*p*+1)^)), for some *c* > 0, such that

(55) $$\begin{array}{c} \gamma \left(f\right)=\sum_{n=0}^{\infty }a_{n}\gamma_{n}, \end{array}$$

where *f*(*z*) = ∑_*n*=0_^*∞*^ *a*_*n*_ *z* ^*n*^ ∈ *M* ^*p*^, with convergence being absolute. Conversely, if {*γ*_*n*_} is a sequence of complex numbers for which

(56) $$\begin{array}{c} \gamma_{n}=O\left(\exp\left({-cn}^{1/(p+1)}\right)\right), \end{array}$$

then (55) defines a continuous linear functional on *M* ^*p*^.

**Corollary 18**

*M* ^*p*^ is an *F*-algebra.

**Proof**

By Theorem 15, *M* ^*p*^ becomes an *F*-space. As *N* ^*p*^ is an *F*-algebra, by Theorem 16, the multiplication is also continuous on *M* ^*p*^. Hence, *M* ^*p*^ is an *F*-algebra.

## 5. Bounded Subsets of *M* ^*p*^

It is proved in Section 4 (Theorem 16) that the spaces *M* ^*p*^ and *N* ^*p*^ coincide and have the same topological structure. Since *N* ^*p*^ and *M* ^*p*^ are not Banach spaces, it is of interest to obtain a characterization of bounded subsets of these spaces in terms of both metrics *d*_*p*_ and *ρ*_*p*_.

Recall that, for a function *f* ∈ *N* ^*p*^, its boundary function f* is defined as the radial limit f*(*e* ^*iθ*^) = lim⁡_*r*→1−_ *f*(*re* ^*iθ*^) which exists for almost every *e* ^*iθ*^ ∈ *𝕋*.

The following result gives a characterization of bounded subsets of *N* ^*p*^( = *M* ^*p*^). Recall that the assertion (i)⇔(iii) is analogous to Theorem 1 in [21] that describes bounded subsets of *N* ^+^.

**Theorem 19**

For given set *L* ⊂ *M* ^*p*^, the following conditions are equivalent:

(i) *L* is a bounded subset of *M* ^*p*^;
(ii) for all *ε* > 0 there exists *δ* > 0 such that
  (57) $$\begin{array}{c} \int_{E}^{}{\left(\log^{+}Mf\left(\theta \right)\right)}^{p}\frac{d\theta }{2\pi }<\varepsilon \;\forall f\in L, \end{array}$$
  for every measurable set *E* ⊂ *𝕋* with the Lebesgue measure |*E* | <*δ*;
(iii) for all *ε* > 0 there exists *δ* > 0 such that
  (58) $$\begin{array}{c} \int_{E}^{}{\left(\log^{+}\left|f^{*}\left(e^{i\theta }\right)\right|\right)}^{p}\frac{d\theta }{2\pi }<\varepsilon \;\forall f\in L, \end{array}$$
  for each measurable set *E* ⊂ *𝕋* with the Lebesgue measure |*E* | <*δ*.

**Proof**

(ii)⇒(iii). It follows that from the obvious inequality |f*(*e* ^*iθ*^)|≤*Mf*(*θ*), *f* ∈ *M* ^*p*^, for almost every *θ* ∈ [0,2*π*].

(iii)⇒(i). Let

(59) $$\begin{array}{c} V=\left\{g\in N^{p}:\;d_{p}\left(g,0\right)<\eta \right\} \end{array}$$

be an arbitrary neighborhood of zero in *N* ^*p*^. Choose sufficiently small *ε* > 0 such that

(60) $$\begin{array}{c} \log^{p}\left(1+\varepsilon \right)+2^{p-1}\log^{p}2\delta +2^{p-1}\varepsilon <\eta^{p}. \end{array}$$

Now it follows that there exists *δ*, 0 < *δ* < *ε*, such that (iii) holds. Choose an *n* ∈ *ℕ* for which 1/*n* < *δ*. Set

(61) $$\begin{array}{c} E_{k}=\left\{e^{i\theta }:\;\theta \in \left[\frac{2\left(k-1\right)\pi }{n},\frac{2k\pi }{n}\right)\right\},\;k=1,2,\ldots ,n. \end{array}$$

Then |*E*_*k*_ | = 1/*n* < *δ*, and thus by (iii) we have

(62) ∫02π(log⁡+⁡|f∗(eiθ)|)pdθ2π =∑k=1n∫Ek<nε‍ ∀f∈L.

By (62) and Chebyshev's inequality, we conclude that for every function *f* ∈ *N* ^*p*^ there exists a measurable set *E*_*f*_ ⊂ *𝕋* depending on *f* such that

(63) $$\begin{array}{c} \left|𝕋\setminus E_{f}\right|<\delta ,\;\;{\left(\log^{+}\left|f^{*}\left(e^{i\theta }\right)\right|\right)}^{p}\leq \frac{n\varepsilon }{\delta }\;\mathrm{on}\;\;E_{f}. \end{array}$$

From (63), we obtain

(64) $$\begin{array}{c} \left|f^{*}\left(e^{i\theta }\right)\right|\leq \exp{\left(\frac{n\varepsilon }{\delta }\right)}^{1/p}=K\left(\delta \right)=K\;\mathrm{on}\;\;E_{f}. \end{array}$$

Choose *α* such that 0 < *α* < *ε*/*δ*. Then using the inequality

(65) $$\begin{array}{c} \log^{p}\left(1+\left|a\right|\right)\leq 2^{p-1}\left({\left(\log^{+}\left|a\right|\right)}^{p}+\log^{p}2\right), \end{array}$$

(60) and (iii), for every *f* ∈ *L*, we obtain

(66) (dp(αf,0))p =∫02πlog⁡p⁡(1+|αf∗(eiθ)|)dθ2π =∫Ef+∫𝕋∖Ef ≤∫Eflog⁡p⁡(1+ε)dθ2π  +2p−1(∫𝕋∖Eflog⁡p⁡2dθ2π+∫𝕋∖Ef(log⁡+⁡|f∗(eiθ)|)pdθ2π) ≤log⁡p⁡(1+ε)+2p−1log⁡p⁡2δ+2p−1ε <ηp.

Therefore, *d*_*p*_(*αf*, 0) < *η*, from which it follows that *αL* ⊂ *V*. Hence, *L* is a bounded subset of *N* ^*p*^.

(i)⇒(ii). Assume that *L* is a bounded subset of *M* ^*p*^. Then for any given *η* > 0 there is a *α*_0_ = *α*_0_(*η*), 0 < *α*_0_ < 1, such that

(67) $$\begin{array}{c} {\left(\rho_{p}\left(\alpha f,0\right)\right)}^{p}=\int_{0}^{2\pi }\log^{p}\left(1+\left|\alpha \right|Mf\left(\theta \right)\right)\frac{d\theta }{2\pi }<\eta^{p} \end{array}$$

for each *f* ∈ *L* and |*α* | ≤*α*_0_. It follows that

(68) ∫02π(log⁡+⁡|α|Mf(θ))pdθ2π<ηpfor  each  f∈L,|α|≤α0.

Since

(69) $$\begin{array}{c} \log^{+}Mf\left(\theta \right)\leq \log^{+}\alpha_{0}Mf\left(\theta \right)+\log\frac{1}{\alpha_{0}}, \end{array}$$

we obtain

(70) $$\begin{array}{c} {\left(\log^{+}Mf\left(\theta \right)\right)}^{p}\leq 2^{p-1}\left({\left(\log^{+}\alpha_{0}Mf\left(\theta \right)\right)}^{p}+{\left(\log\frac{1}{\alpha_{0}}\right)}^{p}\right). \end{array}$$

For given *ε* > 0, choose *η* > 0 satisfying

(71) $$\begin{array}{c} \eta <\frac{\varepsilon^{1/p}}{2}, \end{array}$$

and *α*_0_ = *α*_0_(*η*) satisfying (67) and so also satisfying (68). Next, take *δ* > 0 such that

(72) $$\begin{array}{c} \delta \log^{p}\frac{1}{\alpha_{0}}<\frac{\varepsilon }{2^{p}}. \end{array}$$

Then for each set *E* ⊂ *𝕋* with |*E* | <*δ*, by (68)–(72), for every *f* ∈ *L*, we obtain

(73) ∫E(log⁡+⁡Mf(θ))pdθ2π ≤2p−1(∫E(log⁡+⁡α0Mf(θ))pdθ2π+∫Elog⁡p⁡1α0dθ2π) ≤2p−1ηp+2p−1|E|log⁡p⁡1α0 ≤ε.

Therefore, the condition (ii) of the theorem is satisfied, which concludes the proof.

**Remark 20**

Note that the condition (ii) from Theorem 19 in fact means that the family {(log⁡^+^ *Mf*(*θ*))^*p*^ : *f* ∈ *L*} is uniformly integrable on *𝕋*. The same assertion is also valid for the condition (iii). On the other hand, from the proof of Theorem 19, we see that (ii) implies that the family {(log⁡^+^ *Mf*(*θ*))^*p*^ : *f* ∈ *L*} forms a bounded subset of the space *L* ^1^(*𝕋*); that is, there holds

(74) $$\begin{array}{c} \underset{f\in L}{\mathrm{limsup}}\int_{0}^{2\pi }{\left(\log^{+}Mf\left(\theta \right)\right)}^{p}\frac{d\theta }{2\pi }<+\infty . \end{array}$$

Similarly, it follows from (iii) that the family {(log⁡^+^|f*(*e* ^*iθ*^)|)^*p*^ : *f* ∈ *L*} is bounded in *L* ^1^(*𝕋*).

**Corollary 21**

If *L* is a subset of *M* ^*p*^ for which the family

(75) $$\begin{array}{c} \left\{{\left(\log^{+}\left|f^{*}\left(e^{i\theta }\right)\right|\right)}^{p}:\;f\in L\right\} \end{array}$$

is uniformly integrable, then the family

(76) $$\begin{array}{c} \left\{{\left(\log^{+}\left|f\left(re^{i\theta }\right)\right|\right)}^{p}:\;f\in L,0\leq r<1\right\} \end{array}$$

is also uniformly integrable.

**Proof**

The condition of Corollary 21 and (iii)⇒(ii) of Theorem 19 immediately yield that the family {(log⁡^+^ *Mf*(*θ*))^*p*^ : *f* ∈ *L*} is uniformly integrable on the circle *𝕋*. This fact and the obvious inequality |*f*(*re* ^*iθ*^)|≤*Mf*(*θ*), *f* ∈ *M* ^*p*^, 0 ≤ *r* < 1, for almost every *θ* ∈ [0,2*π*], imply that the family {(log⁡^+^|*f*(*re* ^*iθ*^)|)^*p*^ : *f* ∈ *L*, 0 ≤ *r* < 1} is uniformly integrable.

The following result gives a necessary condition for a subset of *M* ^*p*^( = *N* ^*p*^) to be bounded.

**Theorem 22**

Let *L* be a subset of *M* ^*p*^. If *L* is bounded in *M* ^*p*^, then

(77) $$\begin{array}{c} M_{\infty }\left(r,f\right)\leq K\exp\left(\frac{\omega \left(r\right)}{{\left(1-r\right)}^{1/p}}\right)\;for\;\;each\;\;f\in L, \end{array}$$

where *M*_*∞*_(*r*, *f*) = max⁡_0≤*θ*<2*π*_ | *f*(*re* ^*iθ*^)|, *K* is a positive constant, and *ω*(*r*), 0 ≤ *r* < 1, is a positive continuous function that does not depend on *f* ∈ *L* and for which *ω*(*r*) ↓ 0 as *r* → 1.

**Proof**

By the inequqlity (5.4) from the proof of Theorem 5.2 in [4], for all *f* ∈ *N* ^*p*^, we have

(78) (log⁡+⁡|f(reiθ)|)p ≤12π∫02π1−r21−2rcos⁡⁡(θ−t)+r2(log⁡+⁡|f∗(eit)|)pdt.

As, by the assumption, *L* is a bounded subset of *N* ^*p*^, by Theorem 19 (iii), for all *ε* > 0 there exists *δ* = *δ*(*ε*) > 0, such that

(79) $$\begin{array}{c} \int_{0}^{2\pi }{\left(\log^{+}\left|f^{*}\left(e^{i\theta }\right)\right|\right)}^{p}\frac{d\theta }{2\pi }<\frac{\varepsilon }{2}\;\forall \;f\in L \end{array}$$

and for every measurable set *E* ⊂ *𝕋* with the Lebesgue measure |*E* | <*δ*.

Further, from the proof of (iii)⇒(i) of Theorem 19, we see that for each *f* ∈ *N* ^*p*^ there is a measurable set *E*_*f*_ ⊂ *𝕋* depending on *f* for which

(80) $$\begin{array}{c} \left|𝕋\setminus E_{f}\right|<\delta ,\;\;{\left(\log^{+}\left|f^{*}\left(e^{i\theta }\right)\right|\right)}^{p}\leq \frac{n\varepsilon }{\delta } \end{array}$$

for almost every *e* ^*iθ*^ ∈ *E*_*f*_. From (78)–(80), we obtain

(81) (log⁡+⁡|f(reiθ)|)p=∫Ef+∫Ef≤nεδ+11−rε2,

whence it follows that

(82) $$\begin{array}{c} \left(1-r\right){\left(\log^{+}M_{\infty }\left(r,f\right)\right)}^{p}\leq \frac{\left(1-r\right)n\varepsilon }{\delta }+\frac{\varepsilon }{2}\;. \end{array}$$

Choose a sequence {*ε*_*k*_} of positive numbers such that *ε*_*k*_ ↓ 0. For each *k* ∈ *ℕ*, let *r*_*k*_ > 0 be a number such that

(83) $$\begin{array}{c} \frac{\left(1-r_{k}\right)n\varepsilon }{\delta_{k}}+\frac{\varepsilon_{k}}{2}<\varepsilon_{k}, \end{array}$$

where *ε*_*k*_ = *δ*(*ε*_*k*_) and

(84) $$\begin{array}{c} r_{k-1}<r_{k}<1,\;r_{k}↑1\;\text{as}\;\;k⟶\infty . \end{array}$$

Put

(85) $$\begin{array}{c} \omega_{1}\left(r\right)=\varepsilon_{k}\;\text{for}\;\;r_{k}\leq r<r_{k+1},\;k=1,2,\ldots . \end{array}$$

From (82), (83), and (85) we obtain

(86) $$\begin{array}{c} {\left(\log^{+}M_{\infty }\left(r,f\right)\right)}^{p}\leq \frac{\omega_{1}\left(r\right)}{1-r}\;\forall \;0\leq r<1. \end{array}$$

Since

(87) $$\begin{array}{c} \omega_{1}\left(r\right)⟶0\;\text{as}\;\;r⟶1, \end{array}$$

we conclude that there exists a continuous function *ω*_2_(*r*) satisfying

(88) $$\begin{array}{c} \omega_{1}\left(r\right)\leq \omega_{2}\left(r\right),\;\omega_{2}\left(r\right)↓0\;\;\text{as}\;\;r\to 1. \end{array}$$

Therefore,

(89) $$\begin{array}{c} {\left(\log^{+}M_{\infty }\left(r,f\right)\right)}^{p}\leq \frac{\omega_{2}\left(r\right)}{1-r}\;\text{for each}\;\;0\leq r<1, \end{array}$$

whence by setting

(90) $$\begin{array}{c} \omega \left(r\right)={\left(\omega_{2}\left(r\right)\right)}^{1/p}\;\text{for each}\;\;0\leq r<1, \end{array}$$

we obtain

(91) $$\begin{array}{c} M_{\infty }\left(r,f\right)\leq \exp\left(\frac{\omega \left(r\right)}{{\left(1-r\right)}^{1/p}}\right)\;\forall f\in L. \end{array}$$

This concludes the proof.

**Remark 23**

The condition of Theorem 22 is not a sufficient condition for a set *L* ⊂ *M* ^*p*^ to be bounded. To show this, define

(92) $$\begin{array}{c} f_{n}\left(z\right)=a_{n}z^{n},\;\;a_{n}=\exp\left(\lambda_{n}n^{1/(p+1)}\right), \end{array}$$

where

(93) $$\begin{array}{c} \lambda_{n}=n^{-1/2(p+1)}. \end{array}$$

Then as in the proof of Lemma 1 in [21] it is easy to verify that the set *L* = {*f*_*n*_} ⊂ *M* ^*p*^ satisfies the condition of Theorem 22. Since

(94) $$\begin{array}{c} \log\left|f_{n}^{*}\left(e^{i\theta }\right)\right|=n^{1/2(p+1)}, \end{array}$$

we see that *L* is not bounded in *M* ^*p*^.

**Theorem 24**

There exist bounded subsets of *M* ^*p*^ that are not relatively compact.

**Proof**

Define a sequence {*h*_*n*_} of functions on [0,2*π*] as

(95) $$\begin{array}{c} h_{n}\left(t\right)=1+\sin\left(nt\right),\;t\in \left[0,2\pi \right], \end{array}$$

and set

(96) fn(z)=exp⁡⁡(12π∫02πeit+zeit−zhn(t)dt)=exp⁡⁡(1−izn), z∈𝔻.

Obviously, {*f*_*n*_} ⊂ *N* ^*p*^ and for each measurable set *E* ⊂ *𝕋* we have

(97) $$\begin{array}{c} \int_{0}^{2\pi }h_{n}\left(t\right)dt=2\pi ,\\ 0\leq \int_{E}^{}h_{n}\left(t\right)dt\leq 2\left|E\right|, \end{array}$$

where |*E*| denotes the Lebesgue measure of *E*. From this and Theorem 19, we see that the set *L* = {*f*_*n*_} is bounded in *N* ^*p*^.

Now suppose that *E* is relatively compact. This means that there exists a subsequence {*f*_*n*__*k*_} of {*f*_*n*_} and a function *f* ∈ *N* ^*p*^ such that

(98) $$\begin{array}{c} d_{p}\left({f_{n}}_{k},f\right)⟶0\;\text{as}\;\;k⟶\infty , \end{array}$$

and thus

(99) fnk(z)⟶f(z),uniformly on each closed disk  |z|≤r<1.

Therefore, by (96), it follows that *f*(*z*) ≡ *e* on *𝔻*. On the other hand, from (98), it follows that

(100) $$\begin{array}{c} {f_{n}}_{k}^{*}\left(e^{i\theta }\right)⟶f^{*}\left(e^{i\theta }\right)\;\text{in measure on}\;\;𝕋. \end{array}$$

Therefore,

(101) log⁡+⁡|fnk∗(eiθ)|=1+sin⁡(nkθ)⟶log⁡+⁡|f∗(eiθ)|=1in measure on  𝕋.

This contradiction shows that *L* is not relatively compact in *N* ^*p*^.

## References

[1] Kim HO (1986). On closed maximal ideals of M. *Proceedings of the Japan Academy A*.

[2] Kim HO (1988). On an *F*-algebra of holomorphic functions. *Canadian Journal of Mathematics*.

[3] Privalov II (1941). *Boundary Properties of Analytic Functions*.

[4] Meštrović R, Pavićević Ž (1996). Remarks on some classes of holomorphic functions. *Mathematica Montisnigri*.

[5] Mochizuki N (1989). Algebras of holomorphic functions between *H*
^*p*^ and *N*
_∗_. *Proceedings of the American Mathematical Society*.

[6] Stoll M (1977). Mean growth and Taylor coeffecients of some topological algebras of analytic functions. *Annales Polonici Mathematici*.

[7] Eoff CM (1988). Fréchet envelopes of certain algebras of analytic functions. *Michigan Mathematical Journal*.

[8] Eoff CM (1993). A representation of *N*
_*a*_
^+^ as a union of weighted Hardy spaces. *Complex Variables, Theory and Application*.

[9] Iida Y, Mochizuki N (1998). Isometries of some *F*-algebras of holomorphic functions. *Archiv der Mathematik*.

[10] Meštrović R (1999). *Topological and F-algebras of holomorphic functions [Ph.D. thesis]*.

[11] Meštrović R, Pavićević Ž (2003). Topologies on some subclasses of the Smirnov class. *Acta Scientiarum Mathematicarum*.

[12] Meštrović R, Pavićević Ž (2011). Weakly dense ideals in Privalov spaces of holomorphic functions. *Journal of the Korean Mathematical Society*.

[13] Meštrović R, Subbotin AV (1999). Multipliers and linear functionals for Privalov spaces of holomorphic functions in the disc. *Doklady Akademii Nauk*.

[14] Meštrović R, Šušić J (2013). Interpolation in the spaces *N*
^*p*^(1 < *p* < 1). *Filomat*.

[15] Matsugu Y (2000). Invariant subspaces of the Privalov spaces. *Far East Journal of Mathematical Sciences*.

[16] Duren PL (1970). *Theory of *H*^p^ Spaces*.

[17] Yanagihara N (1973). Multipliers and linear functionals for the class N^+^. *Transactions of the American Mathematical Society*.

[18] Gavrilov VI, Zaharyan VS (1992). Conformal invariance and the characteristic property of boundary values of functions in the class M. *Doklady Akademii Nauk Armenii*.

[19] Nawrocky M (2002). On the Smirnov class defined by the maximal function. *Canadian Mathematical Bulletin*.

[20] Yanagihara N (1973). The containing Fréchet space for the class N^+^. *Duke Mathematical Journal*.

[21] Yanagihara N (1973). Bounded subsets of some spaces of holomorphic functions. *Scientific Papers of the College of General Education, University of Tokyo*.

[22] Dunford N, Schwartz JT (1958). *Linear Operators, Part I: General Theory*.

[23] Rudin W (1973). *Functional Analysis*.

